# Supplementary material for: MetaRibo-Seq measures translation in microbiomes
Source: Nat Commun. 2020 Jun 29;11:3268. doi: 10.1038/s41467-020-17081-z (PMC7324362; doi:10.1038/s41467-020-17081-z)
Supplement: Supplementary file 10 — Supplementary Data 7 [file 41467_2020_17081_MOESM10_ESM.zip › File2/Confidence_High_Taxonomy/347910_out.krona.html]

Javascript must be enabled to view this page.

members
magnitude
magnitudeUnassigned
count
unassigned
taxon
rank

347910\_out

189

superkingdom
189
2

976
189
phylum

class
200643
189

order
189
6
171549

SRS049712\_contig\_number\_contig-100\_1089.118531SRS050974\_contig\_number\_contig-100\_68.69SRS1041145\_contig\_number\_2940SRS140492\_contig\_number\_33674SRS147653\_contig\_number\_6062SRS893341\_contig\_number\_8892

9
815
family

genus
9
816

1
817

SRS1041129\_contig\_number\_210
species

46506

SRS143523\_contig\_number\_201
1
species

1

SRS149244\_contig\_number\_1734
47678
species

species

SRS024388\_contig\_number\_1153SRS054590\_contig\_number\_584SRS1041132\_contig\_number\_5711
246787
3

1
820

SRS020622\_contig\_number\_37
species


SRS075984\_contig\_number\_319
469589
1
species

28116

SRS019968\_contig\_number\_2339
1
species

171
2005525
family

genus
171

SRS017701\_contig\_number\_12825SRS018575\_contig\_number\_7215SRS019068\_contig\_number\_63764SRS020328\_contig\_number\_38373SRS021219\_contig\_number\_13301SRS022609\_contig\_number\_13770SRS022713\_contig\_number\_7548SRS023715\_contig\_number\_25668SRS024132\_contig\_number\_50373SRS024435\_contig\_number\_12784SRS043667\_contig\_number\_5285SRS045244\_contig\_number\_7117SRS046369\_contig\_number\_11962SRS050422\_contig\_number\_28344SRS051610\_contig\_number\_4128SRS052697\_contig\_number\_20210SRS058723\_contig\_number\_6746SRS058770\_contig\_number\_13260SRS063040\_contig\_number\_45504SRS063370\_contig\_number\_571SRS075821\_contig\_number\_7516SRS075878\_contig\_number\_17488SRS077335\_contig\_number\_7989SRS077392\_contig\_number\_38977SRS077641\_contig\_number\_9647SRS078419\_contig\_number\_9002SRS098571\_contig\_number\_23730SRS098717\_contig\_number\_646SRS1041091\_contig\_number\_10902SRS1041134\_contig\_number\_9697SRS1041137\_contig\_number\_14337SRS104311\_contig\_number\_35209SRS1054716\_contig\_number\_7313SRS1055049\_contig\_number\_2151SRS142503\_contig\_number\_13920SRS143722\_contig\_number\_10423SRS144603\_contig\_number\_contig-100\_949.89794SRS145497\_contig\_number\_contig-100\_301.118332SRS147271\_contig\_number\_42395SRS149181\_contig\_number\_24634SRS893231\_contig\_number\_683SRS893252\_contig\_number\_1646SRS893382\_contig\_number\_4594SRS971275\_contig\_number\_41549
375288
44

1
1750560

SRS1041039\_contig\_number\_2209
species

species
2
46503

SRS056519\_contig\_number\_10112SRS098061\_contig\_number\_7914


SRS075021\_contig\_number\_7031SRS076976\_contig\_number\_13437
563193
2
species

species
2

SRS015794\_contig\_number\_7042SRS018351\_contig\_number\_1694
469591


SRS019496\_contig\_number\_9536
1262912
1
species


SRS077849\_contig\_number\_28941SRS1041133\_contig\_number\_18462
2293116
2
species

4

SRS015782\_contig\_number\_18591SRS017521\_contig\_number\_31195SRS045713\_contig\_number\_23SRS048060\_contig\_number\_5696
2293114
species

823

SRS015663\_contig\_number\_36834SRS015890\_contig\_number\_21903SRS015960\_contig\_number\_15168SRS016056\_contig\_number\_7269SRS016095\_contig\_number\_11440SRS016267\_contig\_number\_462SRS016335\_contig\_number\_25857SRS016989\_contig\_number\_contig-100\_1854.89453SRS017103\_contig\_number\_755SRS017191\_contig\_number\_11250SRS017433\_contig\_number\_contig-100\_1945.102666SRS018313\_contig\_number\_985SRS018541\_contig\_number\_4727SRS018623\_contig\_number\_14522SRS018656\_contig\_number\_11817SRS018836\_contig\_number\_19535SRS018888\_contig\_number\_2453SRS018984\_contig\_number\_4853SRS019030\_contig\_number\_16453SRS019178\_contig\_number\_97SRS019267\_contig\_number\_8383SRS019397\_contig\_number\_383SRS019601\_contig\_number\_30069SRS019685\_contig\_number\_7144SRS019787\_contig\_number\_11149SRS020233\_contig\_number\_53247SRS020394\_contig\_number\_contig-100\_957.86139SRS021948\_contig\_number\_3699SRS022071\_contig\_number\_11438SRS023583\_contig\_number\_9850SRS023829\_contig\_number\_87SRS023971\_contig\_number\_21SRS043701\_contig\_number\_9344SRS043768\_contig\_number\_23226SRS045195\_contig\_number\_7917SRS045645\_contig\_number\_25022SRS045826\_contig\_number\_11413SRS047014\_contig\_number\_28972SRS049773\_contig\_number\_18638SRS049900\_contig\_number\_17661SRS049959\_contig\_number\_28513SRS050026\_contig\_number\_12387SRS050752\_contig\_number\_11652SRS050925\_contig\_number\_24363SRS050941\_contig\_number\_13788SRS051882\_contig\_number\_4213SRS052027\_contig\_number\_519SRS052078\_contig\_number\_1208SRS053214\_contig\_number\_21900SRS053398\_contig\_number\_26572SRS053649\_contig\_number\_3586SRS056259\_contig\_number\_3999SRS056273\_contig\_number\_32184SRS056537\_contig\_number\_11653SRS058145\_contig\_number\_3815SRS062701\_contig\_number\_1470SRS064645\_contig\_number\_403SRS064973\_contig\_number\_338SRS065397\_contig\_number\_contig-100\_685.75295SRS065504\_contig\_number\_29099SRS074964\_contig\_number\_1574SRS075398\_contig\_number\_164SRS076804\_contig\_number\_18486SRS077127\_contig\_number\_17832SRS077194\_contig\_number\_32503SRS077454\_contig\_number\_19353SRS077730\_contig\_number\_9961SRS078242\_contig\_number\_31604SRS078665\_contig\_number\_contig-100\_1230.55785SRS097920\_contig\_number\_2664SRS100021\_contig\_number\_12056SRS103987\_contig\_number\_31633SRS104036\_contig\_number\_2711SRS1041036\_contig\_number\_59SRS1041092\_contig\_number\_16097SRS1041095\_contig\_number\_32SRS1041138\_contig\_number\_contig-100\_33.76260SRS1041141\_contig\_number\_11789SRS1041142\_contig\_number\_52SRS1041147\_contig\_number\_2873SRS104327\_contig\_number\_1742SRS104693\_contig\_number\_7929SRS1055038\_contig\_number\_59SRS1055069\_contig\_number\_14217SRS142542\_contig\_number\_8361SRS142599\_contig\_number\_11241SRS142712\_contig\_number\_4270SRS142890\_contig\_number\_20882SRS143148\_contig\_number\_contig-100\_395.150240SRS143342\_contig\_number\_13139SRS143876\_contig\_number\_11680SRS144362\_contig\_number\_6492SRS144506\_contig\_number\_19404SRS146764\_contig\_number\_31288SRS146813\_contig\_number\_3249SRS147614\_contig\_number\_152SRS148196\_contig\_number\_10904SRS148253\_contig\_number\_5330SRS148511\_contig\_number\_11304SRS148784\_contig\_number\_18602SRS148817\_contig\_number\_13272SRS148874\_contig\_number\_16660SRS893253\_contig\_number\_309SRS893256\_contig\_number\_200SRS893259\_contig\_number\_5921SRS893285\_contig\_number\_500SRS893288\_contig\_number\_33SRS893342\_contig\_number\_9454SRS893383\_contig\_number\_35430
109
species

3

SRS097958\_contig\_number\_24480SRS144297\_contig\_number\_1554SRS971276\_contig\_number\_33484
1947048
species


SRS1041090\_contig\_number\_135
1944636
1
species

171551
3
family

genus
836
3

3

SRS019910\_contig\_number\_20939SRS048870\_contig\_number\_21816SRS064557\_contig\_number\_13209
658663
species
